# Inhibition of autophagy and chemokine induction by sphingosine 1-phosphate receptor 1 through NF-κB signaling in human pulmonary endothelial cells infected with influenza A viruses

**DOI:** 10.1371/journal.pone.0205344

**Published:** 2018-10-10

**Authors:** Lan Wang, Hao Jiang, Si-mei Shen, Chun-xia Wen, Zheng Xing, Yi Shi

**Affiliations:** 1 Department of Respiratory and Critical Care Medicine, Jinling Clinical Medical College of Nanjing Medical University, Nanjing, China; 2 Department of Respiratory Medicine, the Affiliated Jiangyin Hospital of Southeast University, Jiangyin, China; 3 Department of Tuberculosis, the Second Affiliated Hospital, Southeast University, Nanjing, China; 4 Medical School and Jiangsu Key Laboratory of Molecular Medicine, Nanjing University, Nanjing, China; 5 Veterinary and Biomedical Sciences, College of Veterinary Medicine, University of Minnesota, Twin Cities, Saint Paul, MN, United States of America; University of Hong Kong, HONG KONG

## Abstract

Endothelial cells have been considered the central regulators of cytokine storm in the respiratory system during influenza virus infection. Studies have found that elevated autophagy could be an essential component of viral pathogenesis in influenza infection. However, few studies have been performed to examine whether autophagy occurs in human pulmonary endothelial cells (HPMECs). In addition, specific mechanisms about how inflammatory responses are regulated in the endothelial cells remain unclear. We hypothesized that infection of influenza A viruses subtypes H1N1 and H9N2 triggered autophagy, which played an important role in the induction of proinflammatory cytokines, both in human lung epithelial A549 cells and in HPMECs. In this report, we showed our evidence that blockage of autophagy significantly inhibited influenza virus-induced proinflammatory responses and suppressed viral replication. Our data indicated that the inhibition of the cytokine response and viral replication was affected by increasing the expression of endothelial sphingosine 1-phosphate receptor 1 (S1PR1), which might be through the regulation of NF-κB signaling. Overexpression of S1PR1 decreased p65 phosphorylation and translocation into the nucleus. Furthermore, we demonstrated that S1PR1 stimulation inhibited Akt-mTOR signaling, which might contribute to activation of autophagy in HPMECs. Thus, our study provides knowledge crucial to better understanding novel mechanisms underlying the S1PR1-mediated attenuation of cytokine amplification in the pulmonary system during influenza virus infection.

## Introduction

Newly emerging and re-emerging infections of influenza A viruses (IAV) have posed considerable threats to public health, in particular the ones of highly pathogenic avian influenza with early exacerbation and dysregulation of innate cellular and cytokine responses, or cytokine storm [1,2,3]. Recent studies on IAV infection have documented a significant association between excessive early immune cell recruitment and poor clinical prognosis [4,5]. Mounting evidence has identified pulmonary endothelial cells as central regulators of the cytokine storm, which challenges the long-standing assumption that alveolar epithelial cells are the main target cell type in viral pathogenesis in influenza [4]. We previously found that specific agonist CYM5442 of sphingosine 1-phosphate receptor 1 (S1PR1) inhibited induction of pro-inflammatory cytokines and chemokines [6]. The endogenous S1P acting on endothelial S1PR1 could be a negative regulator of cytokine amplification [4,6].

Autophagy is an endogenous inhibitory and strictly regulated process, essential to maintain cellular homeostasis by removing damaged organelles, misfolded proteins, and invaded pathogens [7,8]. Autophagy plays an important role in the course of virus infection and host immune responses [9,10]. Accumulating data have revealed that elevated autophagy induced by IAV mediates alveolar epithelial cell death and is important for replication of IAV [11,12,13]. However, to date little has been known about whether autophagy occurs in HPMECs, and if so, whether S1PR1 may have any impact on autophagy upon IAV infection. Here, we provide evidence that IAV not only stimulated proinflammatory cytokines but also induced autophagy both in human lung epithelial A549 cells and in HPMECs. We demonstrated that over-expressed S1PR1 in pulmonary endothelial cells suppressed autophagy, inhibited the inflammatory responses and virus replication, which might be regulated by suppressing NF-κB signaling. Thus, autophagic pathway affected by S1PR1 signaling in the pulmonary endothelial cells could provide a novel therapeutic target for attenuation of mortality and morbidity in influenza infection.

## Materials and methods

### Cells and cell culture

Primary human pulmonary microvascular endothelial cells (HPMECs) and human umbilical vein endothelial cells (HUVECs) obtained from Lonza (Walkersville, CA) were cultured in the Endothelial Cell Medium (ECM) with recommended supplements from the supplier and used in passages 3 to 5. The Madin-Darby canine kidney (MDCK) cell line, human sarcoma HeLa cells, HUVECs were all purchased from American Type Culture Collection (ATCC, Manassas, VA). They were cultured in Dulbecco’s Modified Eagle’s medium (DMEM, Gibco, Gaithersburg, MA) supplemented with 10% fetal bovine serum (FBS, Thermo Fisher, Waltham, MA), penicillin-streptomycin (100U/ml, Thermo Fisher). Human lung epithelial cells A549 were purchased from ATCC, and cultured in RPMI 1640 (Thermo Fisher) with 5% FBS. Cells were incubated in a humidifier incubator at 37°C with 5% CO_2_.

### Antibodies, plasmids and reagents

Primary antibodies for LC3B, Atg5, mTOR, p-mTOR (Ser^2448^), GAPDH, Akt, p-Akt(Thr^308^), p-Akt (Thr^473^), p65, and p-p65 were obtained from Cell Signaling Technology (Danvers, MA). Antibodies for S1PR1 (EDG1), anti-influenza A virus nucleoprotein (NP) as well as FITC- conjugated goat anti-mouse and Cy3-conjugated goat anti-rabbit secondary antibodies were purchased from Abcam (Cambridge, MA). Horseradish peroxidase (HRP)-conjugated secondary antibodies were obtained from Cell Signaling Technology. Lipofectamine 3000 transfection reagent was purchased from Thermo Fisher. Plasmid pEGFP-LC3 was kindly provided by Dr. Jiwu Wei, Nanjing University Medical School, China. Plasmid pS1PR1-PRK5-HA and CYM5442 was described previously [6]. The sequence of an S1PR1-specific siRNA was CGGTCTCTGACTACGTCAA.

### Virus and plasmid transfection

The influenza viruses used in this study were A/Nanjing/108/2009 (H1N1) and A/Hong Kong/2108/2003 (H9N2) as reported in previous studies [6,14]. Experiments were performed in biosafety level 2 containment facilities with the approval of the ethics committee of Jinling Hospital, Nanjing University Medical School (Approval Number: JLYY: 2013021). The viruses were propagated and titrated as described before [6]. HPMECs were transiently transfected with plasmids of pS1PR1-PRK5-HA or pEGFP-LC3 by using Lipofectamine 3000 when the cells reached 50–70% confluence according to the manufacturer’s instructions. Six hours later, the cells were cultured in fresh medium for another 36 hrs, followed by infection with the H1N1 or H9N2 virus at a multiplicity of infection (MOI) of 1 in some experiments.

### Western blot and real-time PCR

Total cell protein was extracted according to the procedures described previously [6]. Protein concentration was measured by bicinchoninic acid assay (BCA) using a commercial kit (Thermo Fisher). The samples were denatured at 95–100°C for 5 min before being loaded onto the SDS-PAGE gel and subsequently transferred on the PVDF membrane (Hercules, Bio-Rad) for western blot analysis. The membranes were incubated in 25 ml of blocking buffer (5% milk in PBST) for 1 hr at room temperature, followed by incubation of primary antibodies (at a dilution recommended in the product datasheet) with gentle agitation overnight at 4°C. After incubation with the HRP-conjugated secondary antibody with gentle agitation for 1 hr at room temperature, the signals were visualized by using Enhancing Chemiluminescence (ECL) System (Amersham Biosciences, Piscataway, NJ). The densities of signals were scanned and analyzed using Image J software for quantitative analysis.

Total RNA was isolated from HPMECs and A549 cells by using Trizol reagent (Invitrogen, Carlsbad, CA) as previously described [6], followed by reverse transcription to synthesize complementary DNA (cDNA) with an RT-PCR kit (Thermo Fisher) according to the supplier’s instruction. Real-time PCR analysis for tumor necrosis factor (TNF)-α, interleukin (IL)-1β, CCL1, IL-6, IL-8, Rantes, CXCL-8, interferon (IFN)-α, IFN-β, Beclin-1, S1PR1, M gene of the H1N1 virus, and housekeeping gene GAPDH was performed by using SYBR Green Master Mix (Thermo Fisher) and analyzed with a ViiA 7 Software. Specific primers used in PCR were listed in Table 1.

**Table 1:** Primer sequences for quantitative real-time PCR.

Target genes	Direction	Sequences
*TNF-*α	Forward	5’TCAACCTCCTCTCTGCCATC3’
	Reverse	5’CCAAAGTAGACCTGCCCAGA3’
*IL-1β*	Forward	5’TGAAATGATGGCTTATTACAGTGG3’
	Reverse	5’GTAGTGGTGGTCGGAGATTCGTAG3’
*IL-6*	Forward	5’CACACAGACAGCCACTCACC3’
	Reverse	5’TTTTCTGCCAGTGCCTCTTT3’
*Rantes*	Forward	5’TACACCAGTGGCAAGTGCTC3’
	Reverse	5’TGTACTCCCGAACCCATTTC3’
*IL-8*	Forward	5’GTTCCACTGTGCCTTGGTTT3’
	Reverse	5’GCTTCCACATGTCCTCACAA3’
*Beclin1*	Forward	5’TGGACAGTTTGGCACAATCAA3’
	Reverse	5’TTCCGTAAGGAACAAGTCGGTAT3’
*CXCL8*	Forward	5’TCAGAGACAGCAGAGCACAC3’
	Reverse	5’GGCAAAACTGCACCTTCACA3’
*Ifn-*α	Forward	5’AAAGAAATGTAAGAAAGCTTTTGATGA3’
	Reverse	5’TACACTTTGGCTCAGGACTCATTT3’
*Ifn-β*	Forward	5’TGGGAGGCTTGAATACTGCCTCAA3’
	Reverse	5’TCCTTGGCCTTCAGGTAATGCAGA3’
*S1PR1*	Forward	5’CAATTTCTCATGCCGCACAG3’
	Reverse	5’AGCTGGAAGATCGAAAGTCCG3’
*H1N1 M*	Forward	5’GGTGTCACTAAGCTATTCAA3’
	Reverse	5’CAAAAGCAGCTTCTGTGGTC3’
*GAPDH*	Forward	5’ACAGTCAGCCGCATCTTCTT3’
	Reverse	5’ACGACCAAATCCGTTGACTC3’

### Immunofluorescence staining and confocal microscopy

HPMECs and A549 cells were seeded onto microscopy glass coverlips in 24-well plates. Cells were infected with the H1N1 or H9N2 viruses at an MOI of 1. At 24 hrs post infection, the cells were washed with PBS and fixed with 4% paraformaldehyde for 15 min and permeabilized in 0.1% Triton X-100 for 3 min before incubation with specific antibodies with appropriate titrations. Nuclei were stained and determined by DAPI for 1 min before observation under a confocal fluorescence microscope. The cells expressing GFP-LC3 through transfection of pGFP-LC3 were infected with the H1N1 or H9N2 viruses in some experiments. Autophagy was assessed by fluorescence of pGFP-LC3 in transfected/infected cells by fluorescence microscopy.

To detect the viral protein, LC3 puncta, or NF-κB in HPMECs, cells were incubated with antibodies for influenza viral NP, LC3 or NF-κB p65 subunit for 2 hrs, followed by further incubation with FITC- or Cy3-conjugated secondary antibodies and confocal fluorescence microscopy.

### Statistical analysis

All data are presented as means±SEM. Statistical analyses were performed with student’s *t* tests or ANOVA followed by Bonferroni’s post-hoc tests. Statistical tests were performed with GraphPad Prism 5.0 (GraphPad Software, La Jolla, CA). P values of less than 0.05 were considered statistically significant.

## Results

### Autophagosomes were observed in IAV-infected human lung epithelial and endothelial cells

**Fig 1:**
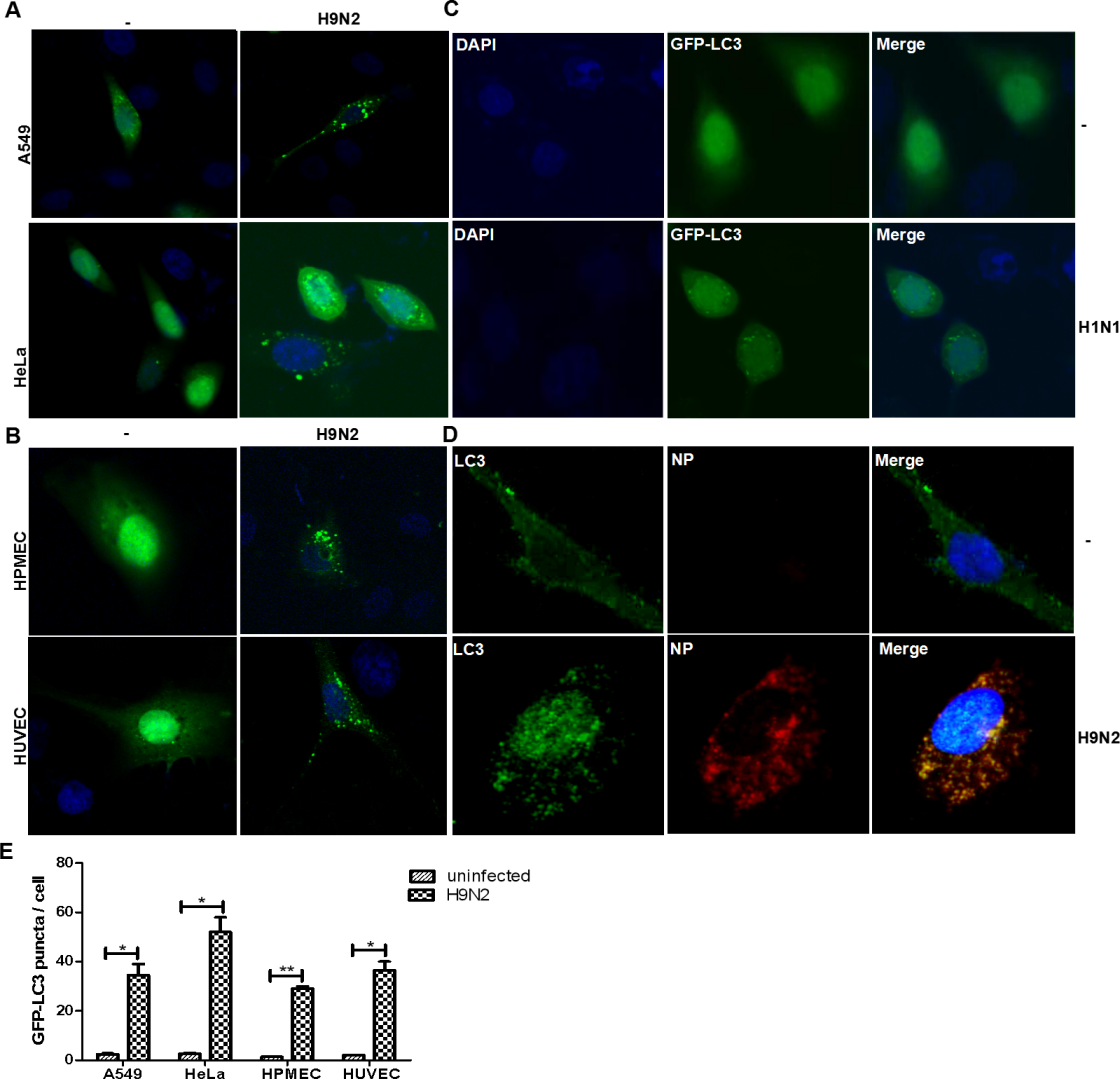
Autophagsomes were formed and accumulated after IAV infection in various cell types including endothelial cells. The cells were transiently transfected with plasmids expressing GFP-LC3 and thereafter infected with IAV H9N2 or H1N1. The cells were observed for GFP-LC3 distribution in the cytoplasm by confocal fluorescence microscopy at 24 hrs post infection. DAPI was used for nuclear staining. Scale bar, 20μm. (**A**) A549 and HeLa cells infected with H9N2; (**B**) HPMECs and HUVECs infected with H9N2; (**C**) HeLa cells infected with an H1N1 IAV; (**D**) The presence of viral NP and LC3 in H9N2-infected HPMECs. Scale bar, 20μm; (**E**). Quantitative analyses of autophagosomes in infected and non-infected cells of various types (** p<0.05; ***p<0.01).

To investigate whether autophagy occurred in human lung endothelial cells as well as epithelial cells upon IAV infection, we first infected A549 and HeLa cells, both transiently transfected with plasmids expressing GFP-LC3, with an influenza virus H9N2. In uninfected cells, GFP-LC3 was distributed diffusely throughout the cytoplasm as previously described [15], but GFP-LC3 formed punctate granules clustered around the perinuclear region in infected cells (Fig 1A). We tried two types of endothelial cells, HPMECs and HUVECs. Both HPMECs and HUVECs were transfected with pGFP-LC3 prior to the infection with IAV H9N2. Likewise, LC3-formed granules, likely the autophagosomes, were observed in HPMECs and HUVECs indistinguishably (Fig 1B), indicating that autophagy occurred in IAV-infected endothelial cells. Influenza virus subtypes appeared to have no difference in initiating autophagy. In HPMECs infected with H9N2, we also detected the presence of influenza viral NP proteins together with LC3. We found that the punctate granules of LC3 appeared only in NP-positive cells (Fig 1D), indicating that it was the influenza infection triggered the autophagic process in the endothelial cells.

We tried a different subtype of influenza virus, the H1N1, to infect HeLa cells which were firstly transfected with pGFP-LC3. The identical punctate granules of LC3 appeared in the cells infected with the H1N1 virus and almost no autophagosomes were observed in the uninfected cells (Fig 1C). Quantitative comparison of the autophagosomes between infected and non-infected cells was shown in Fig 1E.

**Fig 2:**
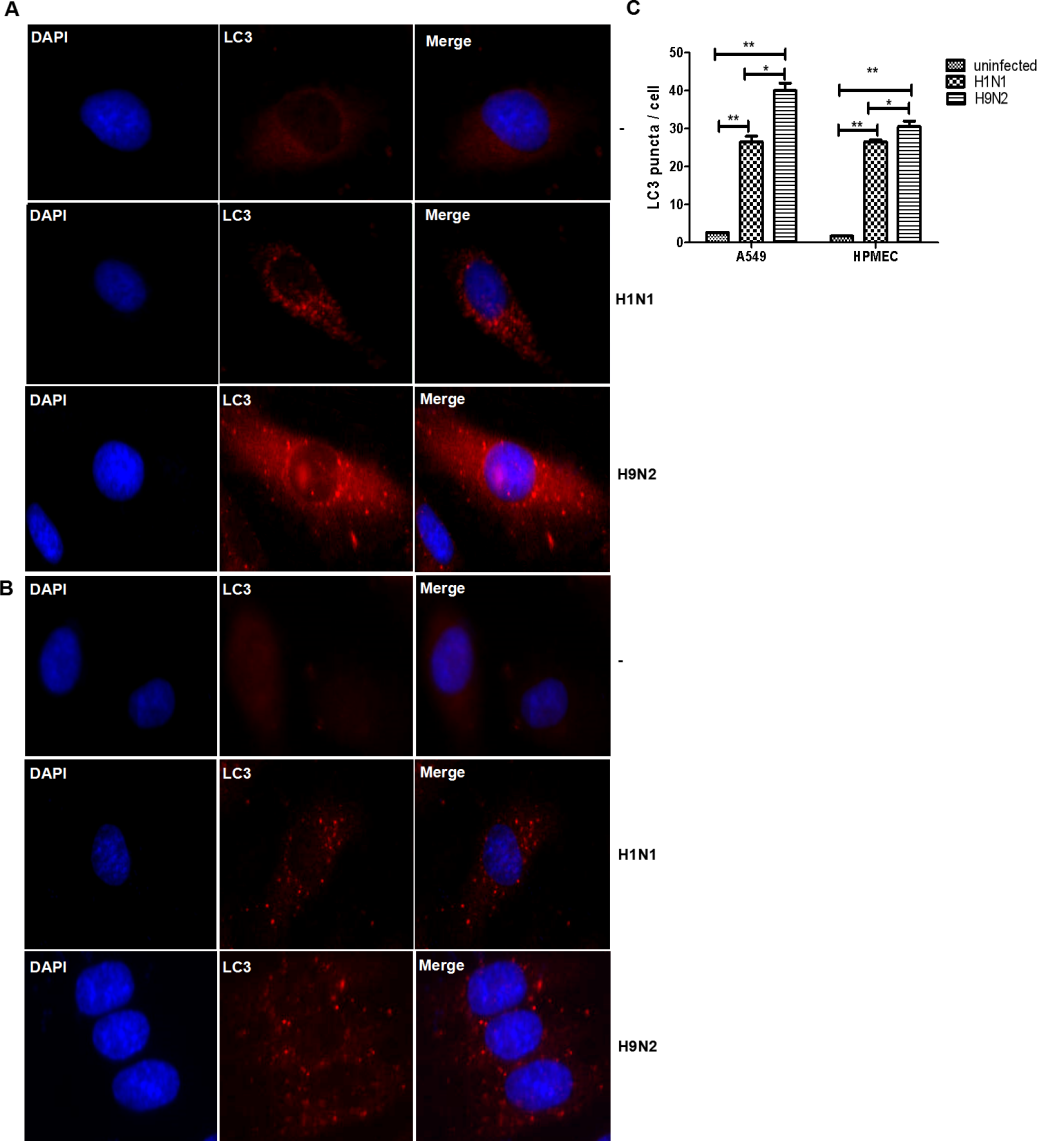
IAV H1N1 and H9N2 subtypes induced autophagy in A549 cells and HPMECs. Cells were infected with IAV subtypes H1N1 or H9N2 at an MOI of 1. Anti-LC3 antibody was used to stain the cells for immunofluorescence microscopy at 24 hrs post infection. (**A**) A549 cells; (**B**) HPMECs. Scale bar, 10μm; (**C**) Quantitative analyses of autophagosomes in infected and non-infected A549 and HPMECs (** p<0.05; ***p<0.01).

To further confirm the autophagy initiated in HPMECs and A549 cells upon IAV infection, we tried to detect the endogenous LC3 by using an anti-LC3 antibody to stain the cells infected with IAV and subsequently examined the LC3 distribution and morphology. As shown in Fig 2, the infection with the H1N1 and H9N2 could induce the characteristic distributional and morphological change of LC3, formed the LC3 punctate granules in the perinuclear area of the cytoplasm, and induced autophagy, effectively in the lung epithelial A549 cells (Fig 2A) and HPMECs (Fig 2B). Quantitative comparison of the autophagosomes between infected and non-infected cells was shown in Fig 2C.

**Fig 3:**
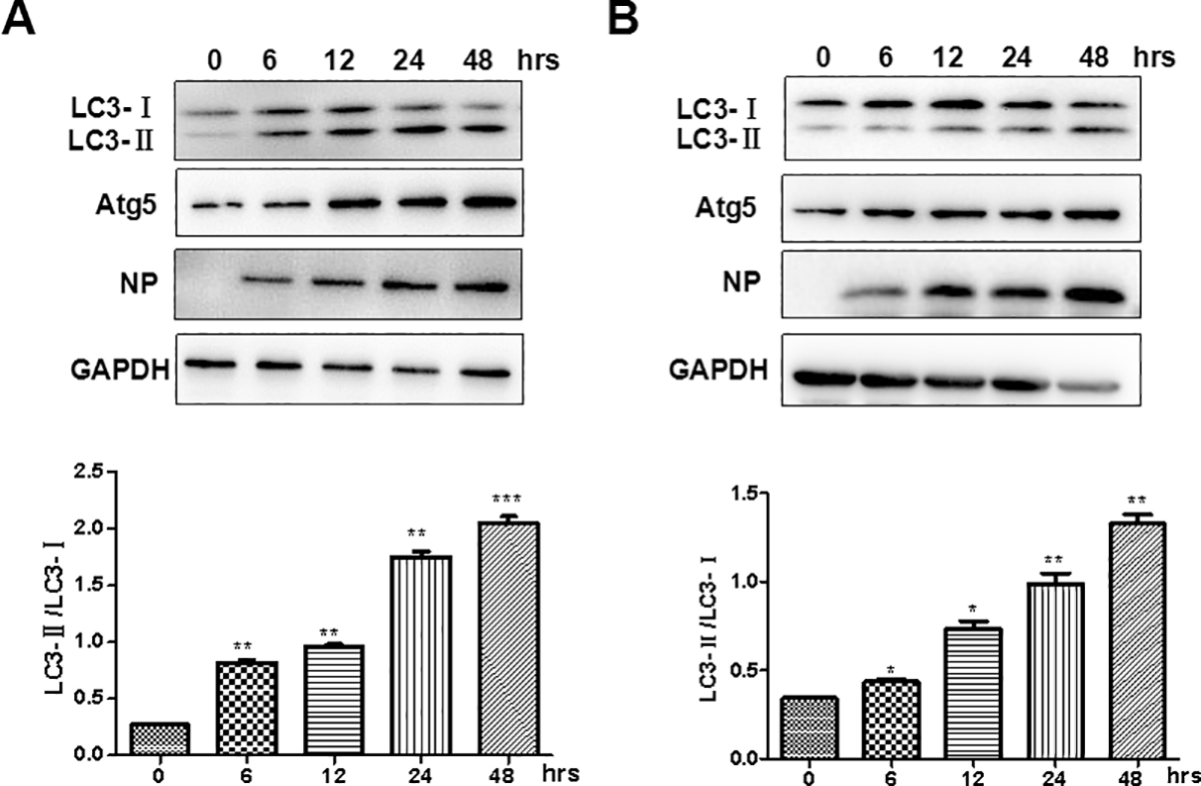
IAV triggered autophagy in HPMECs. HPMECs were infected with either IAV H1N1 (**A**) or H9N2 (**B**) at an MOI of 1. At 24 hrs post infection, cell lysates were prepared and analyzed for SDS-PAGE, followed by a western blot analysis with antibodies specific for LC3-I/II, NP and Atg 5. Quantitative analyses of the LC3-II/LC3-I ratios in either H1N1 or H9N2-infected cells were shown in the lower panels (** p<0.05; ***p<0.01).

LC3 was also analyzed in the infected cells by a western blot analysis to detect LC3-I and LC3-II proteins. Cell lysates were prepared from infected cells harvested at various time points for the analysis. In addition to the increased levels of Atg5 and NP detected in the cells, endogenous LC3-I and LC3-II were clearly detected and distinguished with molecular weights of 16kD and 14kD, respectively, in the infected cells (Fig 3). The densities of protein signals were quantified by scanning individual signal bands for calculation and compared as shown in the lower panels.

### Pro-inflammatory and antiviral cytokine induction in the endothelial cells after IAV infection

**Fig 4:**
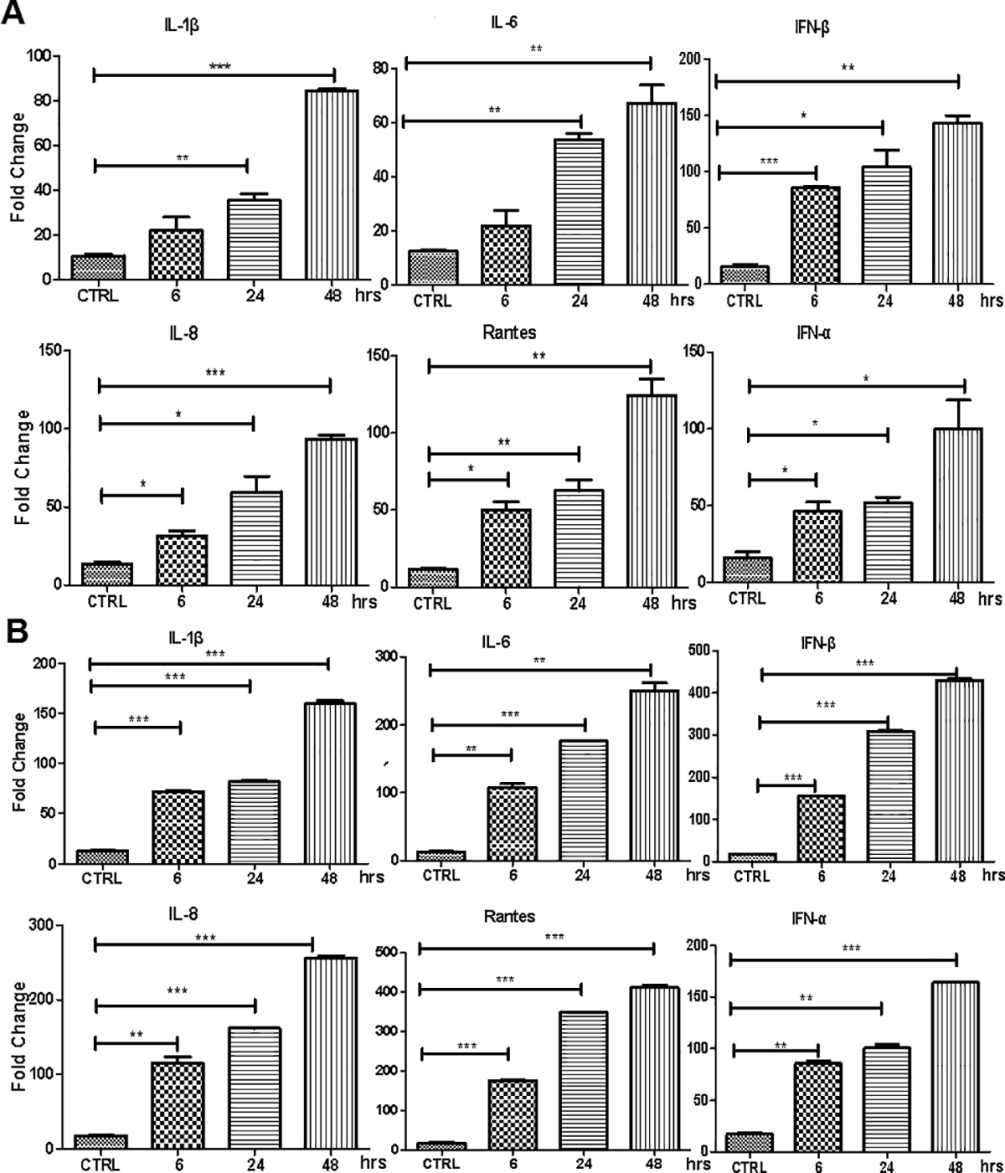
Profiling of the proinflammatory mRNA transcripts in HPMECs infected with IAV H1N1 and H9N2. Cultured HPMECs were infected with H1N1, H9N2 or were mock infected. Total RNA were prepared at indicated time points and cDNA synthesized for analysis of fold change of the selected genes by real-time RT-PCR. The experiments have been repeated for three times. Statistical analyses were performed with ANOVA. * P<0.05; ** P<0.01; *** P<0.001. (**A**) H1N1-infected cells; (**B**) H9N2-infected cells.

We next examined proinflammatory responses in endothelial cells infected with IAV H9N2 and H1N1. HPMECs grown in 12-well plates were infected with the H9N2 and H1N1 viruses, respectively, and total RNA were prepared from the infected cells at the indicated time points. Real-time RT-PCR was performed to analyze the mRNA transcript changes of selected host genes involved in proinflammatory responses. As shown in Fig 4, the transcripts of pro-inflammatory cytokines and chemokines increased apparently in a time-dependent manner after infection with H1N1 or H9N2. Significant inductions occurred at 24 hrs for IL-1β and IL-6 or as early as 6 hrs for RANTES and IL-8 in H1N1-infected cells (Fig 4A). In H9N2-infected cells, all four types of cytokines were induced significantly at 6 hrs post infection (Fig 4B). Moreover, more robust induction of the cytokines appeared in H9N2-infected HPMECs. In addition, antiviral IFN-α and IFN-β were induced significantly as well in either H1N1 or H9N2-infected endothelial cells (Fig 4).

### S1PR1 suppressed autophagy induced by IAV infection in endothelial cells

**Fig 5:**
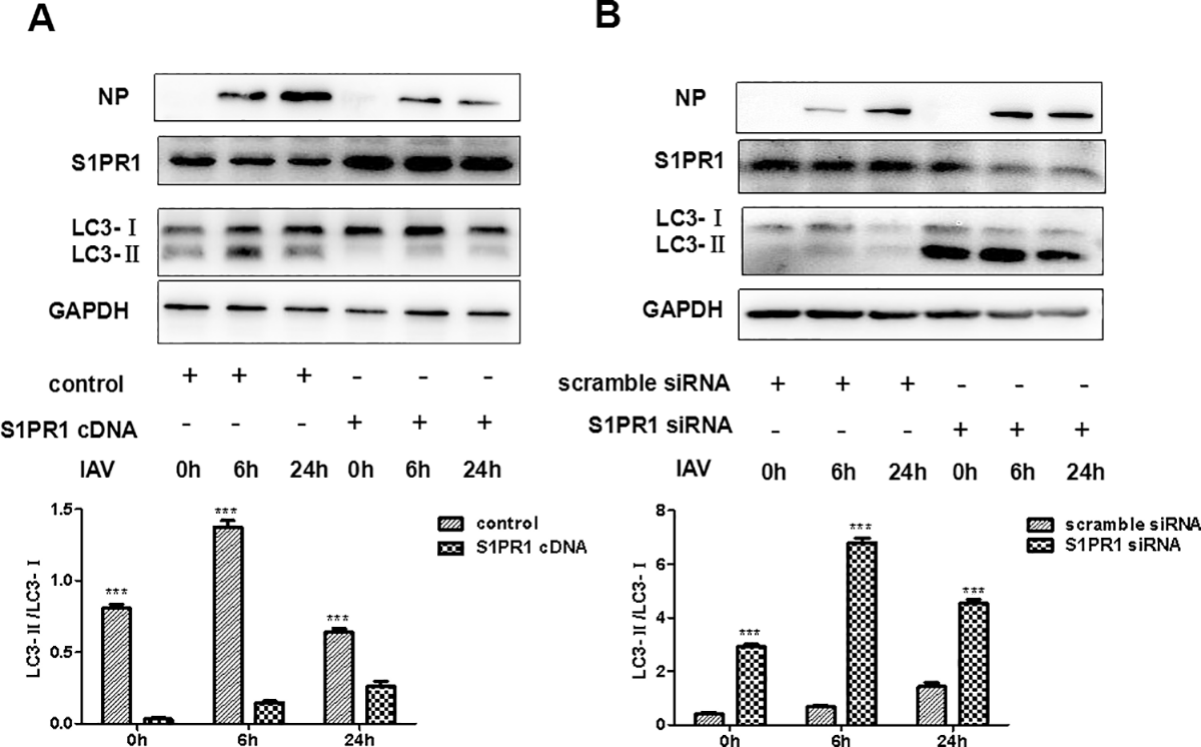
Effect of overexpression or silence of S1PR1 on autophagy in endothelial cells infected with IAV. HPMECs were transfected with plasmids overexpressing S1PR1 (**A**), or with either S1PR1-specific or scramble siRNA (**B**), prior to IAV H1N1 infection. Cell lysates were prepared at 0, 6, and 24 hrs post infection for western blot analyses with specific antibodies for S1PR1, NP, and LC3-I/II. The imaging densities of LC3-I and LC3-II were scanned and the ratios of LC3-II/LC3-I were calculated and exhibited in the lower panels with statistical analyses (*** p<0.01).

We were interested in understanding whether S1PR1 may be involved in the regulation of autophagy in IAV-infected HPMECs. HPMECs were transfected with a plasmid encoding cDNA of S1PR1 for overexpression. We also explored that the HPMECs were transfected with an S1PR1-specific siRNA to knockdown the expression of S1PR1, or scramble siRNAs as a control. Efficiency of the siRNA interference was verified by a western blot analysis (data now shown). To examine the impact of S1PR1 on autophagy in the endothelial cells, siRNAs were used to transfect HPMECs, which were subsequently infected with the IAV H1N1. Cell lysates were prepared from the infected and treated cells at various time points for detection of protein levels of LC3-I, LC3-II, and S1PR1 by a western blot analysis. As shown in Fig 5, LC3-I and LC3-II were detected in the infected cells and the relative ratios of LC3-II/LC3-I significantly decreased in the cells with overexpressed S1PR1 (Fig 5A) but increased in the cells with silenced S1PR1 (Fig 5B). The densities of the LC3-II and LC3-I were scanned and the ratios of LC3-II/LC3-I were quantified as shown in the lower panels. Our results indicated that, when S1PR1 decreased in quantity, the production of LC3-II increased, resulting in an activation of autophagy in infected endothelial cells. In contrast, while S1PR1 increased by overexpression, production of LC3-II decreased and autophagy was suppressed.

### Inhibition of autophagy impaired the viral replication and inflammatory response via the S1PR1 signaling pathway

**Fig 6:**
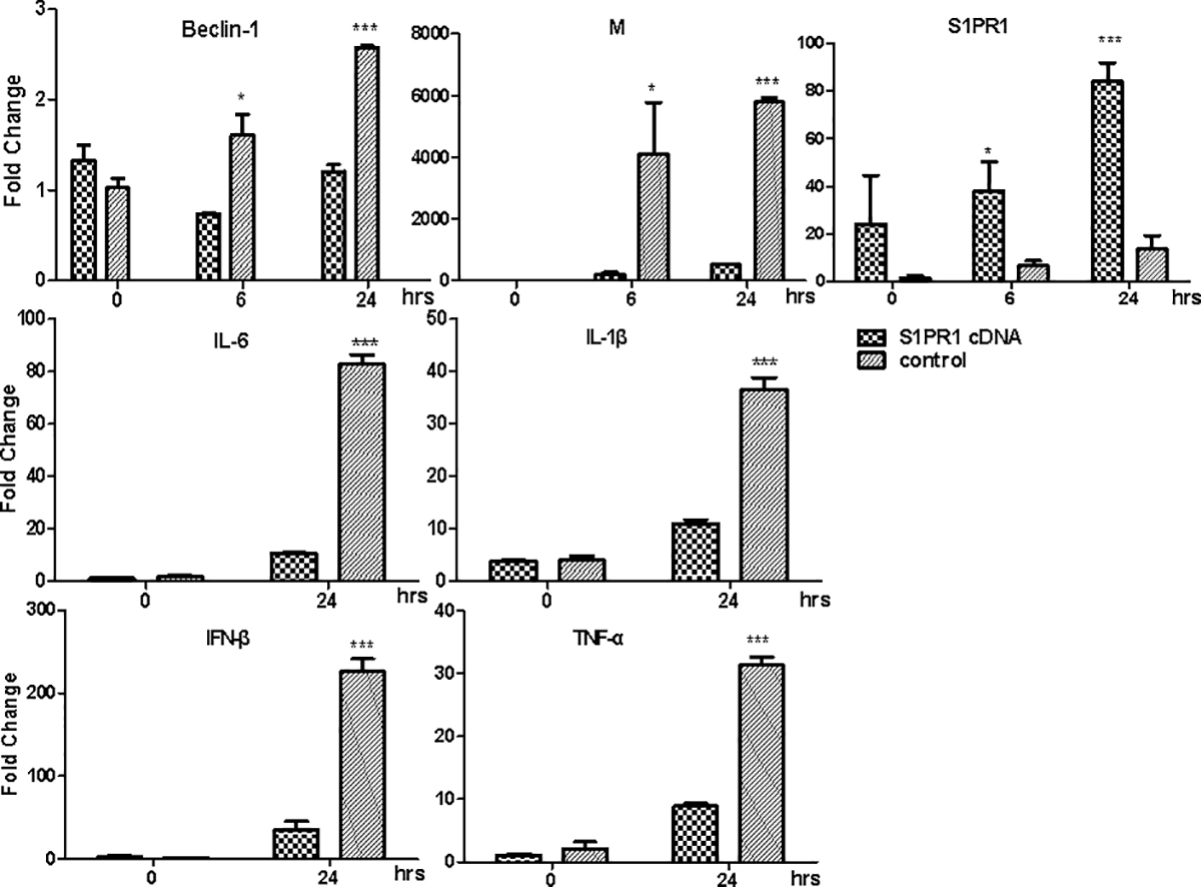
Overexpression of S1PR1 led to suppression of autophagy and viral replication in IAV-infected HPMECs. HPMECs were transfected with the plasmid expressing HA-tagged S1PR1 prior to IAV H1N1 infection. Total RNA were prepared from the infected at indicated time points for real-time RT-PCR to measure the mRNA transcripts with primers specific for Beclin-1, viral matrix, and S1PR1 genes (top panels), or IL-1β, IL-6, TNF-α, and IFN-β (middle and bottom panels). Statistical analyses were performed with Student *t* test (*p<0.05; *** p<0.001).

To examine whether autophagy affects viral replication and host inflammatory responses in IAV-infected endothelial cells, we infected HPMECs with the IAV H1N1 and total RNA was prepared from the infected cells for real-time RT-PCR. We aimed to measure the mRNA transcripts of the viral matrix (M), Beclin-1, and proinflammatory cytokine genes. HPMECs were transfected with the plasmid expressing HA-tagged S1PR1 or with the S1PR1-specific siRNA, prior to the viral infection. As shown in Fig 6 and S1 Fig, we found that, in comparison with the cells treated with the S1PR1 siRNA, overexpressed S1PR1 led to down-regulation of Beclin-1 as well as M genes, and caused the suppression of autophagy and virus replication, suggesting that S1PR1 might be involved in the viral replication through the regulation of autophagy. Our study also demonstrated that activation of the S1PR1 signaling might have blunted the induction of proinflammatory cytokines (TNF-α, IL-1β, IL-6) and type I IFN (IFN-β). Taken these data together, we hypothesized that the S1PR1 signaling may inhibit the inflammatory responses by suppressing autophagy, and the IAV-induced autophagy could be involved in the host proinflammatory responses in HPMECs.

### S1PR1 inhibited Akt-mTOR and NF-κB signaling in IAV-infected HPMECs

**Fig 7:**
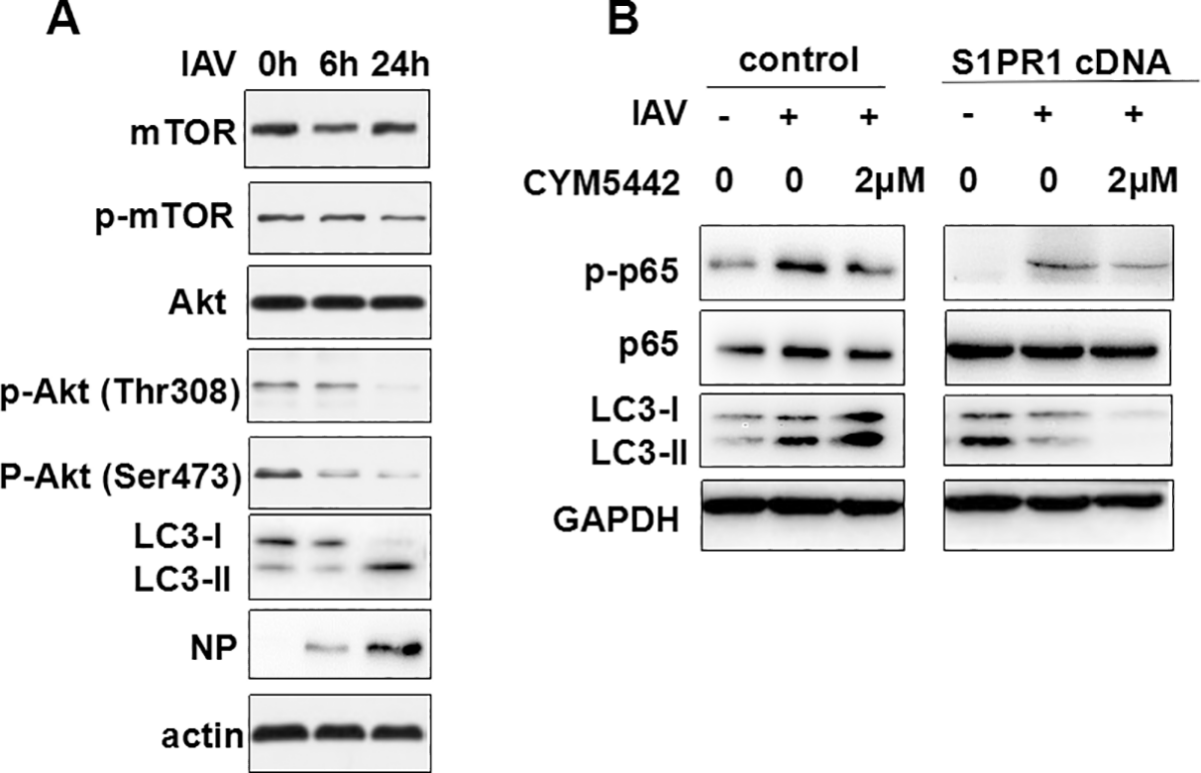
Akt-mTOR and NF-κB signaling were inhibited in the endothelial cells infected with IAV. (**A**) HPMECs were infected with H9N2 at an MOI of 1. Cell lysates were prepared at various time points and subjected to western blot analysis with indicated antibodies. (**B**) HPMECs were transfected with the plasmid expressing S1PR1, or/and treated with 2μM of CYM5442, followed by infection with the IAV H9N2. At 24 hrs post infection, cell lysates were prepared and subjected to SDS-PAGE and western blot analysis with indicated antibodies.

Since Akt-mTOR signaling is also involved in the regulation of autophagy, we next examined whether Akt-mTOR signaling pathway was affected in IAV-infected endothelial cells. HPMECs were infected with the IAV H9N2 and cell lysates were prepared at various time points post infection for western blot analysis. Our results showed that the IAV infection reduced phosphorylation of Akt at both Thr 308 and Ser 473, which might lead to dephosphorylation of mTOR at Ser 2448, at 24 hrs in particular, and upregulation of autophagy with increased LC3II/LC3-I ratios at 24 hrs post infection as shown in Fig 7A. The data suggested that the induction of autophagy could be mediated by inhibition of Akt-mTOR signaling in IAV-infected HPMECs.

**Fig 8:**
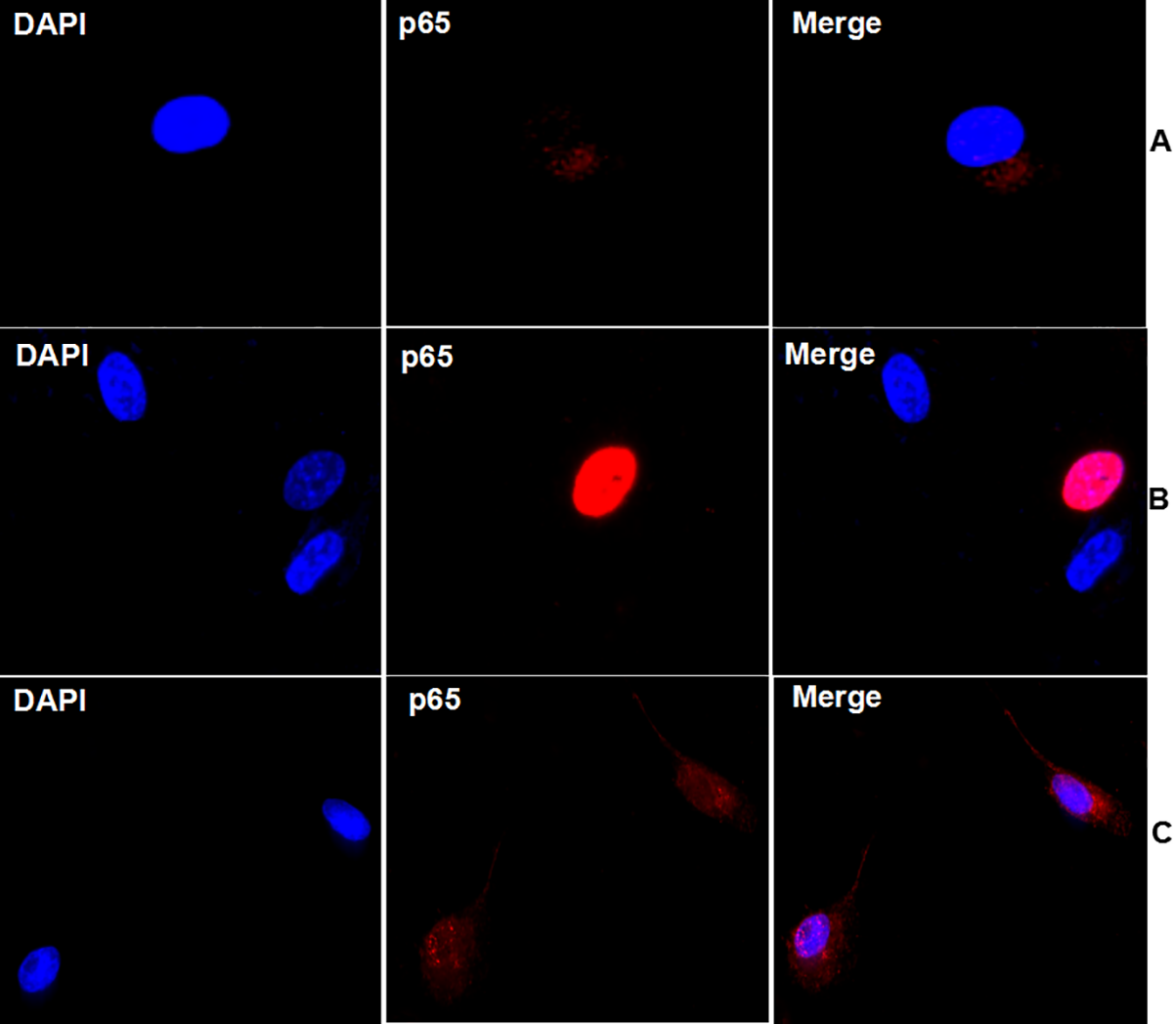
Activation of S1PR1 blocked p65 from translocation into the nucleus. HPMECs were transfected with the plasmid expressing HA-tagged S1PR1 and treated with CYM5442 prior to the IAV H9N2 infection. At 24 hrs post infection, the cells were stained with an anti-NF-κB p65 antibody and subsequently a secondary antibody. Nuclear translocation of NF-κB p65 was examined by confocal microscopy. (**A**) non-infected HPMECs; (**B**) H9N2-infected HPMECs; (**C**) HPMECs, transfected with S1PR1 plasmid and treated with CYM5442, and subsequently infected with the IAV H9N2. Scale bar, 50μm.

Previous studies have shown that NF-κB signaling played a critical role in the proinflammatory responses induced by infection of highly pathogenic avian influenza virus such as the H5N1 virus [16]. We further examined whether overexpression or activation of S1PR1 affected the NF-κB signaling and autophagy in H9N2 virus-infected HPMECs. The cells were pre-treated with or without CYM5442, an agonist of S1PR1, or transfected with a plasmid encoding cDNA of S1PR1, prior to the IAV H9N2 infection. As shown in Fig 7B, infection of the H9N2 virus increased the phosphorylation of NF-κB p65 in comparison to non-infected and non-treated cells. We also observed the increased translocation of p65 into the nucleus in after infection (Fig 8), which indicates that NF-κB signaling was activated in HPMECs. However, when S1PR1 was overexpressed or the cells were treated with CYM5442, the p65 phosphorylation decreased. When the cells were both transfected with the plasmid and treated with CYM5442, IAV-induced autophagy decreased with significantly lowered LC3II/LC3-I ratios along with much decreased phosphorylation (Fig 7B) and blocked nuclear translocation (Fig 8) of NF-κB p65 in HPMECs. Taken together, our data suggest that S1PR1 plays a critical role in the attenuation of proinflammatory response and inhibition of autophagy by suppressing NF-κB signaling in IAV-infected HPMECs.

## Discussion

In the present study, we have demonstrated that influenza A virus was able to trigger autophagy in human pulmonary endothelial cells (HPMECs). We showed that activation of the endothelial S1P1 signaling led to suppression of autophagy, which could result in the decreased induction of proinflammatory cytokines and chemokines and reduced virus replication. It appeared that IAV promoted autophagy by deregulating Akt-mTOR signaling in HPMECs, and our data indicated that S1PR1 might inhibit autophagy and chemokine production through NF-κB signaling. The data shown in this study could provide novel insights into understanding about the regulatory role of endothelial S1PR1 in influenza infection.

S1P is a crucial regulator of a diverse range of cellular processes, including cell trafficking, infections, and inflammation through its binding with high affinity to one of the five S1P receptors (S1PRs), which belong to G-protein-coupled receptor family [17,18,19]. S1P/S1PR1 signaling has been implicated in viral pathogenesis in influenza infection. It was shown previously that endogenous S1P acting on the S1P1 receptor could effectively attenuate production of pro-inflammatory cytokines and chemokines in endothelial cells that serve as central orchestrators of cytokine storm during influenza virus infection [4].

Previous studies have showed that H1N1 or H5N1 viruses-triggered inflammatory responses required the involvement of autophagy and inhibition of autophagy ameliorated lung inflammation [12,16,20]. Autophagy may be a key mechanism for inflammatory responses induced in alveolar epithelial cells and macrophages infected by influenza virus [21]. Autophagy is also important in regulating the immune responses of dendritic cells (DCs) during an H1N1 virus infection [22]. However, the role of autophagy-mediated inflammation has not been elucidated yet in influenza virus-infected HPMECs. In our study, autophagosome accumulation was observed in HPMECs as well as human pulmonary microvascular endothelial cells. Inhibition of autophagy in endothelial cells also blunted the production of pro-inflammatory cytokines, indicating that an association between endothelial autophagy and influenza pathogenesis may occur in pulmonary endothelium.

The roles of autophagy in viral replications could be complex. Although autophagy can protect cells from harmful stimuli and promote cell survival, some pathogens such as influenza viruses could usurp autophagy to benefit their replication [11,23]. Inhibition of autophagy decreases virus yield in both MDCK and A549 cells infected with either H1N1 or H9N2 [11]. In this study, we demonstrated that overexpression of S1PR1 led to suppression of autophagy and decreased viral replication, suggesting that an effective replication of influenza viruses may depend on the autophagic pathway in human pulmonary endothelial cells. Moreover, we found that the IAV H9N2 virus suppressed the activation of the Akt-mTOR signaling, which may have resulted in a significant increase of autophagosome accumulation. This result agrees with a previous report that some viruses including an IAV H5N1 have been found to be involved in the induction of autophagic cell death through inhibition of mTOR signaling [24]. Mechanistically, inhibition of the mTOR signaling triggers autophagy while mTOR signaling may be associated with viral protein accumulation [23].

We also consider that a direct cross-talk may exist between autophagy and NF-κB signaling which could open up new insights into the role of autophagy in diverse biological processes [25]. Autophagy was found to promote NF-κB activation and nuclear translocation, which is required for viral RNA synthesis during IAV infection [23,26]. Moreover, inhibition of NF-κB signaling dramatically attenuated autophagy [16]. Our findings in this study suggest that activation of S1PR1 may also be related to inactivation of NF-κB signaling, subsequently decreasing autophagy and viral replication in HPMECs. Further studies are warranted in order to fully understand the functions of S1PR1 in the regulation of NF-κB signaling and autophagy *in vivo*.

In conclusion, our study has demonstrated that influenza virus infections could lead to upregulation of autophagy possibly by deregulating Akt-mTOR signaling in HPMECs and endothelial S1PR1 may inhibit autophagy and cytokine induction, which may be regulated through NF-κB signaling. Although the mechanism needs to be further elucidated, our findings have further broaden our understanding of influenza pathogenesis, implicating S1PR1 signaling and autophagic pathways in the pulmonary endothelial cells, which could become a potential anti-influenza therapeutic target in the future.

## Supporting information

S1 Fig Knockdown of S1PR1 with siRNA led to upexpression of autophagy and increased virus replication. HPMECs were transfected with S1PR1-specific or scramble siRNA, followed by infection with H1N1, and treated with 2μM of CYM5442. mRNA transcript levels of viral matrix gene (M), Beclin-1, and S1PR1 genes (top panels), or IL-1β, IL-6, TNF-α, and IFN-β (middle and bottom panels) were determined by quantitative PCR at indicated time points. Student *t* test, *, P<0.05; ***, P<0.001.
(TIF)
