# Supplementary figures and images for: Inhibition of autophagy and chemokine induction by sphingosine 1-phosphate receptor 1 through NF-κB signaling in human pulmonary endothelial cells infected with influenza A viruses
Source: PLoS One. 2018 Oct 10;13(10):e0205344. doi: 10.1371/journal.pone.0205344 (PMC6179250; doi:10.1371/journal.pone.0205344)

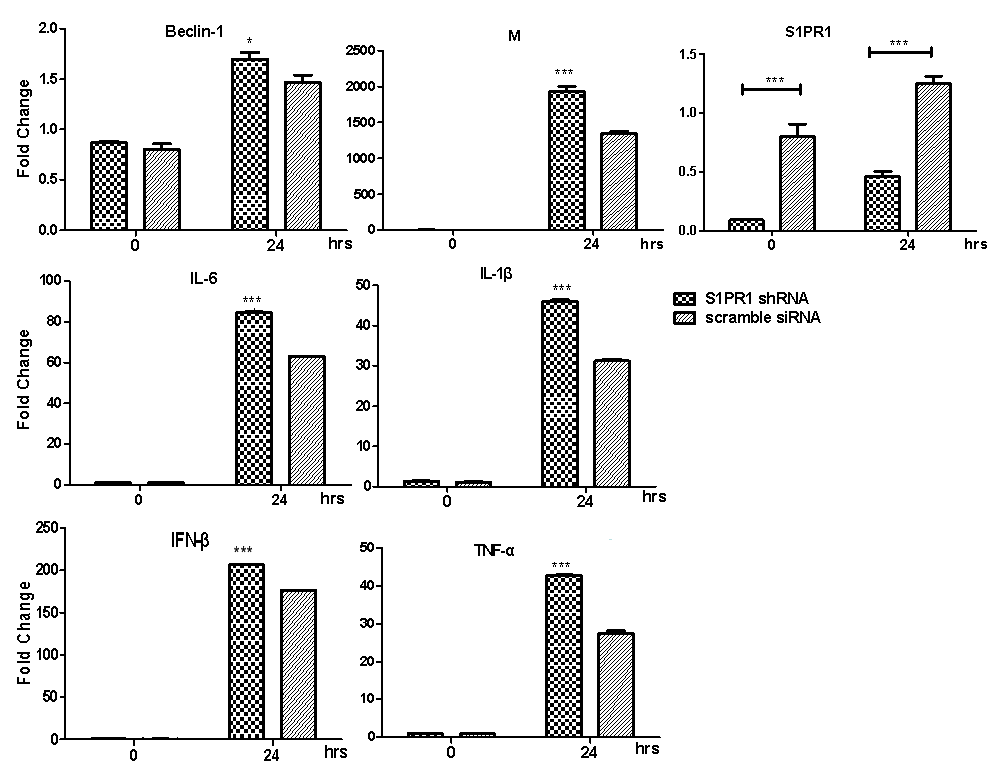

Supplement: S1 Fig — HPMECs were transfected with S1PR1-specific or scramble siRNA, followed by infection with H1N1, and treated with 2μM of CYM5442. mRNA transcript levels of viral matrix gene (M), Beclin-1, and S1PR1 genes (top panels), or IL-1β, IL-6, TNF-α, and IFN-β (middle and bottom panels) were determined by quantitative PCR at indicated time points. Student t test, *, P<0.05; ***, P<0.001. (TIF) [file pone.0205344.s001.tif]
